# Using Machine Learning to Predict Cognitive Decline in Older Adults From the Chinese Longitudinal Healthy Longevity Survey: Model Development and Validation Study

**DOI:** 10.2196/67437

**Published:** 2025-04-30

**Authors:** Hao Ren, Yiying Zheng, Changjin Li, Fengshi Jing, Qiting Wang, Zeyu Luo, Dongxiao Li, Deyi Liang, Weiming Tang, Li Liu, Weibin Cheng

**Affiliations:** 1 Institute for Healthcare Artificial Intelligence Application, The Affiliated Guangdong Second Provincial General Hospital of Jinan University, No. 466 Xingangzhong Road, Haizhu District, Guangzhou, 510317, China, 86 13929587059; 2 Faculty of Data Science, City University of Macau, Macao SAR, China; 3 The Affiliated Traditional Chinese Medicine Hospital, Guangzhou Medical University, Guangzhou, China; 4 School of Public Health, Guangdong Pharmaceutical University, Guangzhou, China; 5 Hainan International College, Minzu University of China, Beijing, China; 6 Guangdong Women and Children Hospital, Guangzhou, China; 7 Institute for Global Health and Infectious Disease, University of North Carolina at Chapel Hill, Chapel Hill, NC, United States; 8 University of North Carolina at Chapel Hill Project-China, Guangzhou, China; 9 College of Computing, City University of Hong Kong, Hong Kong SAR, China

**Keywords:** older adults, cognitive decline, Alzheimer disease, machine learning, blood biomarkers, disease history, Mini-Mental State Examination, MMSE, Chinese Longitudinal Healthy Longevity Survey, CLHLS

## Abstract

**Background:**

Cognitive impairment, indicative of Alzheimer disease and other forms of dementia, significantly deteriorates the quality of life of older adult populations and imposes considerable burdens on families and health care systems worldwide. The early identification of individuals at risk for cognitive impairment through a convenient and rapid method is crucial for the timely implementation of interventions.

**Objective:**

The objective of this study was to explore the application of machine learning (ML) to integrate blood biomarkers, life behaviors, and disease history to predict the decline in cognitive function.

**Methods:**

This approach uses data from the Chinese Longitudinal Healthy Longevity Survey. A total of 2688 participants aged 65 years or older from the 2008‐2009, 2011‐2012, and 2014 Chinese Longitudinal Healthy Longevity Survey waves were included, with cognitive impairment defined as a Mini-Mental State Examination (MMSE) score below 18. The dataset was divided into a training set (n=1331), an internal test set (n=333), and a prospective validation set (n=1024). Participants with a baseline MMSE score of less than 18 were excluded from the cohort to ensure a more accurate assessment of cognitive function. We developed ML models that integrate demographic information, health behaviors, disease history, and blood biomarkers to predict cognitive function at the 3-year follow-up point, specifically identifying individuals who are at risk of experiencing significant declines in cognitive function by that time. Specifically, the models aimed to identify individuals who would experience a significant decline in their MMSE scores (less than 18) by the end of the follow-up period. The performance of these models was evaluated using metrics including accuracy, sensitivity, and the area under the receiver operating characteristic curve.

**Results:**

All ML models outperformed the MMSE alone. The balanced random forest achieved the highest accuracy (88.5% in the internal test set and 88.7% in the prospective validation set), albeit with a lower sensitivity, while logistic regression recorded the highest sensitivity. SHAP (Shapley Additive Explanations) analysis identified instrumental activities of daily living, age, and baseline MMSE scores as the most influential predictors for cognitive impairment.

**Conclusions:**

The incorporation of blood biomarkers, along with demographic, life behavior, and disease history into ML models offers a convenient, rapid, and accurate approach for the early identification of older adult individuals at risk of cognitive impairment. This method presents a valuable tool for health care professionals to facilitate timely interventions and underscores the importance of integrating diverse data types in predictive health models.

## Introduction

Alzheimer disease (AD), the most prevalent form of dementia, is a progressive condition primarily characterized by memory loss [1]. It is estimated that approximately 50 million people worldwide are currently living with AD [2]. This condition not only deteriorates the quality of life for older adult individuals but also imposes significant burdens on families and health care systems, especially as the global population continues to age [3-6]. Mild cognitive impairment (MCI) is recognized as an intermediary stage between normal aging and the more severe cognitive decline observed in dementia. Early detection of MCI through diagnostic tools, such as magnetic resonance imaging (MRI), can facilitate timely interventions aimed at reducing the risk of progression to AD [7]. However, cognitive impairment, which encompasses a broader spectrum of decline, including MCI, is a critical concern that requires early identification. Predicting cognitive impairment, including MCI and other forms of decline, is essential for implementing preventive strategies and improving long-term health outcomes. Therefore, identifying individuals at potential risk for cognitive impairment is crucial.

While MRI and cerebrospinal fluid biomarkers, such as amyloid β, are significant indicators of AD [8], we aim to develop an algorithm that allows for the precise identification of individuals at risk using a more convenient and rapid approach, without relying on complex analyses like MRI or cerebrospinal fluid biomarkers. Recent studies have established a correlation between blood biomarkers and various factors, including diseases such as hypertension, diabetes, heart disease, and cerebrovascular disease, and lifestyle behaviors such as smoking, physical activity, and living conditions, with cognitive impairment [9,10]. High-dimensional data analysis has proven effective in capturing features that are crucial for identifying health issues [11,12]. Hence, the incorporation of these relevant factors into a sophisticated model allows for a practical application both in clinical settings and at home, enabling doctors or families to use existing data to train and monitor patients or older adult individuals, thereby identifying high-risk groups for further treatment and intervention.

In recent years, machine learning (ML), a branch of artificial intelligence, has been increasingly used for the prediction of disease outcome [13,14]. Unlike traditional methods that rely heavily on statistical significance, ML leverages algorithms to process existing factors and develop optimized models [15]. While there has been significant research into understanding the pathogenesis and influencing factors of MCI through ML, most of these studies have primarily focused on imaging techniques such as MRI [16]. Although MRI is a powerful diagnostic tool, its high cost and the inconvenience it poses limit its practicality for widespread use. In contrast, cognitive function assessments, such as the Mini-Mental State Examination (MMSE), offer a more accessible and feasible option for large-scale population screenings [17,18].

In this study, we used follow-up data from the Chinese Longitudinal Healthy Longevity Survey (CLHLS) collected during the years 2008‐2009, 2011‐2012 and 2014, encompassing a total of 2688 participants. Cognitive impairment was classified based on the MMSE scores, with a cutoff point set at 18, to determine cognitive status after 3 years. We included routine blood indices, lifestyle behaviors, and disease history from the baseline data in the ML model for training, aiming to predict the occurrence of cognitive impairment.

## Methods

### Study Participants

The cohort for this study is selected from CLHLS, which is a comprehensive longitudinal survey co-orchestrated by Peking University and the China Aging Science Research Center [19,20]. The survey targets Chinese seniors aged 65 years or older and includes detailed information about their living conditions, socioeconomic status, and health profiles [21]. The CLHLS initiates its baseline survey in 1998 and subsequently conducts follow-up surveys at regular intervals, with the relevant cohorts for this study being those from 2008‐2009, 2011‐2012, and 2014. The CLHLS study maintains ethical standards with approval from the Research Ethics Committee of Peking University (IRB00001052-13074), and all participants or their legal proxies provide written informed consent.

The initial samples from these specified years consist of 8418, 6066, and 3441 participants, respectively. Baseline subjects were screened by MMSE, and cognitive impairment was defined as an MMSE score <18 points [22]. The participants without missing data on MMSE scores at baseline and follow-up while with biomarker data were included in this study. This results in a narrowed-down research sample, with 602 participants from the 2008‐2009 cohort, 1263 from 2011‐2012, and 1116 from 2014, leaving a final sample size of 2688 subjects. Among the remaining participants, those from the 2008‐2009 and 2011‐2012 waves (n=1664) were further divided into a training set (n=1331) and an internal test set (n=333). The 1024 participants from the 2014 wave were used as a prospective validation set.

### Predictors

Demographic predictors include age, gender, and BMI, calculated as weight in kilograms divided by height in meters squared (kg/m^2^). Data on life behaviors and disease history are collected from the CLHLS questionnaire. Life behaviors account for living status (living alone or not), current smoking and drinking habits, exercise practices, marital status, and overall activity ability and mental health. Activity ability is assessed through activities of daily living (ADL) and instrumental activities of daily living (IADL). ADL was assessed by 6 indicators, including bathing, dressing, toileting, indoor transfer, continence, and eating. If all 6 items can be completely self-care, it means that daily life activities can take care of themselves. If one or more items cannot be completely self-care, it means the daily life activities cannot take care of themselves completely (0=normal, 1=disability) [23,24]. IADL was assessed by 8 indicators, including visiting neighbors, going shopping, cooking a meal, washing clothing, walking continuously for 1 km at a time, lifting a weight of 5 kg, continuously crouching and standing up 3 times, and taking public transportation. If 8 items can be completed independently, such as the evaluation method of ADL, it means the instrumental daily life activities can be completed by themselves (0=normal or 1=disability) [23,25,26].

The development of the model in this study encompasses 3 categories of predictors derived from the baseline survey data: biomarkers, life behaviors, and disease history. The set of biomarkers comprises both routine blood examination indices and plasma biochemical examination indices. Routine blood indices include white blood cell count, red blood cell count, hemoglobin, erythrocyte hematocrit, erythrocyte mean corpuscular volume, erythrocyte mean corpuscular hemoglobin, erythrocyte mean corpuscular hemoglobin concentration, platelet count, plateletcrit, mean platelet volume, lymphocyte count, percentage of lymphocytes, and platelet distribution width. The plasma biochemical indices include high-density lipoprotein cholesterol (HDL), uric acid, plasma creatinine, glucose, triglyceride, total cholesterol (CHO), high-sensitivity c-reactive protein, malondialdehyde (MDA), and superoxide dismutase activity.

Mental health evaluation incorporates 7 questions, with 4 positively framed inquiries (regarding optimism, neatness, decision-making, and happiness relative to youth) and 3 negatively framed ones (concerning fear, loneliness, and feelings of decreased self-worth with age). Responses are scored on a scale, with higher scores correlating with poorer mental health. Disease history captures the presence or absence of hypertension, diabetes, heart disease, stroke, cancer, and arthritis. These multifaceted predictors collectively contribute to the ML model, providing a comprehensive profile for the assessment of cognitive impairment risk.

### Data Preprocessing and Model Configuration

The data preprocessing phase involved addressing missing values and mitigating class imbalance to ensure a robust foundation for model training. Missing values in the dataset were imputed using the mean of the respective columns, which ensured completeness and preserved the statistical properties of the data [27]. In order to address the issue of class imbalance, the SMOTE (Synthetic Minority Over-Sampling Technique) was applied to the training set. SMOTE effectively generated synthetic samples for the minority class, enhancing the model’s ability to learn from the imbalanced data distribution [28].

The selection of ML models was driven by a desire to compare different algorithms’ performance and assess their robustness in dealing with imbalanced datasets. We chose 5 widely used algorithms: random forest (RF) [29], Extreme Gradient Boosting (XGBoost) [30], logistic regression [31], support vector machines (SVM) [32], and balanced random forest (BRF) [33]. These models were selected for their varied approaches to classification and their effectiveness in handling different types of data. RF and XGBoost are ensemble models that excel in handling high-dimensional data and capturing nonlinear relationships. Logistic regression and SVM are classic algorithms for binary classification, with SVM known for its ability to handle high-dimensional spaces effectively. The addition of BRF was made specifically to address the class imbalance issue in the RF model. The main difference between RF and BRF is how they handle class imbalance.

Each model was configured as follows:

- XGBoost (XGBClassifier): a learning rate of 0.03 and a maximum depth of 4 were chosen for the XGBoost model, with class weights incorporated to address class imbalance.
- Logistic regression: configured with a maximum of 1000 iterations and class weights set to “balanced” to account for class imbalance.
- SVM: probability estimation was enabled and class weights were balanced to ensure fairness across experiments.
- BRF: class weights were set to “balanced” to address the class imbalance within the dataset.

### Outcomes

The assessment of cognitive function among participants was conducted using the MMSE, administered at both baseline and during follow-up sessions. The MMSE encompasses evaluations across various cognitive domains including orientation, registration, attention and calculation, recall, and language abilities, with a maximum achievable score of 30 points. In this study, the primary outcome for the ML model is the determination of cognitive impairment, defined as an MMSE score of less than 18 points at follow-up.

### Model Construction, Evaluation, and Interpretation

The construction of each model followed a systematic approach to ensure rigorous evaluation and validation. Initially, the CLHLS 2008‐2009 and CLHLS 2011‐2012 datasets were merged. From this combined dataset, 1331/1664 (80%) of the data was randomly allocated as the training set, while the remaining 333/1664 (20%) was reserved as an internal test set for model evaluation. And then, the CLHLS 2014 dataset was used as a prospective validation set, with the model training based exclusively on the merged data from CLHLS 2008‐2009 and CLHLS 2011‐2012. Standard performance metrics—including accuracy, sensitivity, specificity, and the area under the receiver operating characteristic curve—were calculated to assess model effectiveness. All statistical analyses are conducted using Python (version 3.8; Python Software Foundation)

SHAP (Shapley Additive Explanations) [34] is a method for interpreting the output of ML models by assigning a contribution value to each feature, allowing us to understand the impact of individual predictors on a model’s decision. In our study, SHAP was used to explain the predictive model for cognitive impairment in older adult individuals. SHAP values decompose the model’s prediction into individual contributions from each feature, making it possible to attribute the output to the various risk factors in a transparent and interpretable manner.

### Ethical Considerations

The CLHLS was approved by the Duke University Institutional Review Board (Pro00062871) and the Peking University Biomedical Ethics Committee (IRB00001052–13074). All participants provided written informed consent. The data used in this study were deidentified to protect participant privacy and confidentiality. No compensation wasprovided to participants.

## Results

### Participant Characteristics

**Figure 1.**
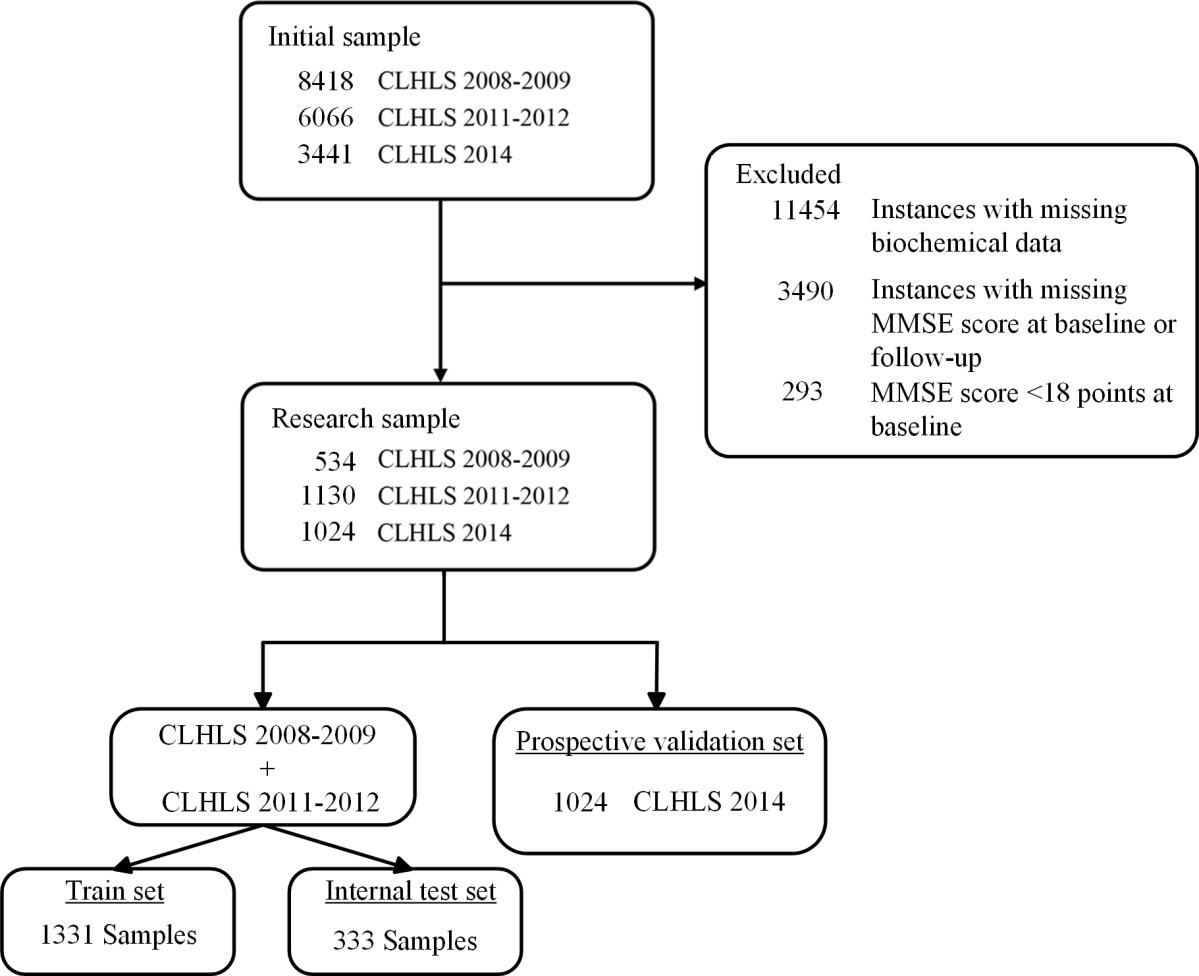
Flow diagram of the subject selection. CLHLS: Chinese Longitudinal Healthy Longevity Survey; MMSE: Mini-Mental State Examination.

Table 1 summarizes the baseline characteristics of the 2688 participants included in this study, stratified into 3 subsets: the training set (n=1331), the internal test set (n=333), and the prospective validation set (n=1024). Overall, the mean age was 79.73 (SD 10.97) years and slightly over half of the participants were male (53.24%). The mean BMI was 21.81 (SD 3.8 kg/m^2^). Most participants lived with others (79.49%), did not smoke (77.79%), or drink (80.96%) at present, and had normal ADL (95.02%). Additionally, more than half (55.28%) showed abnormal mental health status, while 51.45% were married and living with a spouse. Several biochemical indicators were measured. The mean white blood cell count was 5.85 (SD 1.74×10^9^)/L, red blood cell count was 4.64 (SD 1.68×10^12^)/L, and hemoglobin was 131.56 (SD 30.20) g/L. Participants had a mean HDL level of 1.31 (SD 0.38) mmol/L, and other biomarkers (eg, uric acid, creatinine, glucose, triglycerides, CHO, high-sensitivity c-reactive protein, MDA, and superoxide dismutase) were also assessed. Regarding disease history, hypertension was most prevalent (27.18%), followed by arthritis (11.31%), heart disease (8.01%), stroke (6.29%), and diabetes (2.75%). Cancer was reported by 0.42% of participants. Detailed distributions of these characteristics across the training, internal test, and prospective validation sets are presented in Table 1. A detailed flow diagram of participant inclusion and exclusion is provided in Figure 1.

**Table 1.** Characteristics of study subjects at baseline.

Predictors			Overall (n=2688)	Train set (n=1331)	Internal test set (n=333)	Prospective validation set (n=1024)
Demographics and life behaviors						
	Age, year, mean (SD)		79.73 (10.97)	80.24 (11.43)	79.69 (11.6)	79.73 (10.07)
	Gender, n (%)					
		Male	1431 (53.24)	685 (51.47)	173 (51.95)	573 (55.96)
		Female	1257 (46.76)	646 (48.53)	160 (48.05)	451 (44.04)
	BMI, kg/m^2^, mean (SD)		21.81 (3.8)	21.51 (3.86)	21.32 (3.67)	22.31 (3.64)
	Living alone, n (%)					
		Yes	543 (20.51)	267 (20.37)	62 (18.9)	214 (21.21)
		No	2105 (79.49)	1044 (79.63)	266 (81.1)	795 (78.79)
	Smoke at present, n (%)					
		Yes	593 (22.21)	317 (23.94)	72 (21.82)	204 (20.08)
		No	2077 (77.79)	1007 (76.06)	258 (78.18)	812 (79.92)
	Drink at present, n (%)					
		Yes	509 (19.04)	265 (19.98)	57 (17.17)	187 (18.42)
		No	2164 (80.96)	1061 (80.02)	275 (82.83)	828 (81.58)
	Exercise at present, n (%)					
		Yes	2134 (81.08)	1056 (81.23)	267 (81.65)	811 (80.7)
		No	498 (18.92)	244 (18.77)	60 (18.35)	194 (19.3)
	ADL^a^, n (%)					
		Normal	2479 (95.02)	1232 (93.97)	313 (95.72)	934 (96.19)
		Disability	130 (4.98)	79 (6.03)	14 (4.28)	37 (3.81)
	IADL^b^, n (%)					
		Normal	1593 (59.53)	742 (55.87)	198 (59.64)	653 (64.27)
		Disability	1083 (40.47)	586 (44.13)	134 (40.36)	363 (35.73)
	Marital status, n (%)					
		Married and living with spouse	1370 (51.45)	654 (49.32)	165 (49.55)	551 (54.88)
		Others	1293 (48.55)	672 (50.68)	168 (20.45)	453 (45.12)
	Mental health, n (%)					
		Normal	1122 (44.72)	542 (43.78)	139 (44.84)	441 (45.89)
		Abnormal	1387 (55.28)	696 (56.22)	171 (55.16)	520 (54.11)
Biomarkers, mean (SD)						
	WBC^c^, 10^9^/L		5.85 (1.74)	5.63 (1.76)	5.64 (1.89)	6.19 (1.61)
	RBC^d^, 10^12^/L		4.64 (1.68)	4.82 (2.11)	4.98 (1.9)	4.3 (0.56)
	HGB^e^, g/L		131.56 (30.2)	129.98 (26.56)	132.23 (22.69)	133.36 (36)
	HCT^f^, %		36.31 (15.05)	33.77 (18.01)	34.25 (18.1)	40.07 (7.23)
	MCV^g^, fL		93.95 (10.24)	93.51 (12.24)	92.94 (9.76)	94.81 (7.03)
	MCH^h^, pg		29.46 (7.24)	28.38 (5.25)	28.16 (5.75)	31.24 (9.22)
	MCHC^i^, g/L		312.18 (42.25)	304.34 (48.89)	301.38 (54.41)	325.58 (18.74)
	PLT^j^, 10^9^/L		207.32 (99.24)	216.44 (109.67)	213.75 (111.53)	193.65 (77.22)
	PCT^k^, %		0.27 (3.29)	0.33 (4.42)	0.2 (0.1)	0.18 (0.06)
	MPV^l^, fL		9.54 (5.14)	9.43 (2.87)	9.97 (6.53)	9.55 (6.65)
	LYMPH^m^, 10^9^/L		14.56 (15.81)	23.13 (15.58)	21.28 (15.48)	2.03 (1.25)
	LYM%^n^, %		19.55 (16.81)	10.49 (14.48)	11.59 (15.01)	33.06 (9.32)
	PDW^o^, fL		15.47 (4.37)	14.86 (2.38)	14.87 (1.99)	16.44 (6.27)
	HDL^p^, mmol/L		1.31 (0.38)	1.26 (0.37)	1.24 (0.38)	1.41 (0.37)
	UA^q^, umol/L		291 (85.39)	285.55 (85.47)	288.51 (87.31)	298.83 (84.13)
	CRE^r^, mmol/L		81.36 (24.19)	82.49 (25.97)	81.68 (22.2)	79.81 (22.28)
	GLU^s^, mmol/L		5.06 (2.05)	4.83 (2.29)	4.85 (1.87)	5.42 (1.71)
	TG^u^, mmol/L		1.29 (0.95)	1.22 (0.95)	1.44 (1.24)	1.34 (0.82)
	CHO^u^, mmol/L		4.34 (1.17)	4.06 (1.18)	3.94 (1.22)	4.84 (0.95)
	CRPHS^v^, mg/L		4.17 (18.46)	5.46 (23.21)	6.15 (29.35)	2.49 (5.29)
	MDA^w^, nmol/ml		5.64 (3.4)	5.43 (2.76)	5.2 (2.63)	5.93 (4.04)
	SOD^x^, IU/mL		55.53 (10.33)	55.53 (11.9)	53.34 (11.61)	56.04 (8.13)
Disease history, n (%)						
	Hypertension					
		Yes	716 (27.18)	316 (24.09)	88 (26.91)	312 (31.36)
		No	1918 (72.82)	996 (75.91)	239 (73.09)	683 (68.64)
	Diabetes					
		Yes	73 (2.75)	31 (2.34)	11 (3.33)	31 (3.11)
		No	2577 (97.25)	1292 (97.66)	319 (96.67)	966 (96.89)
	Heart disease					
		Yes	212 (8.01)	91 (6.9)	28 (8.54)	93 (9.29)
		No	2436 (91.99)	1228 (93.1)	300 (91.46)	908 (90.71)
	Stroke					
		Yes	167 (6.29)	77 (5.82)	20 (6.08)	70 (6.97)
		No	2490 (93.71)	1247 (94.18)	309 (93.92)	934 (93.03)
	Cancer					
		Yes	11 (0.42)	3 (0.23)	1 (0.3)	7 (0.73)
		No	2588 (99.58)	1301 (99.77)	329 (99.7)	958 (99.27)
	Arthritis					
		Yes	300 (11.31)	183 (13.86)	55 (16.72)	62 (6.18)
		No	2352 (88.69)	1137 (86.14)	274 (83.28)	941 (93.82)

a ADL: activities of daily living.

b IADL: instrumental activities of daily living.

c WBC: white blood cell count.

d RBC: red blood cell count.

e HGB: hemoglobin.

f HCT: erythrocyte hematocrit.

g MCV: erythrocyte mean corpuscular volume.

h MCH: erythrocyte mean corpuscular hemoglobin.

i MCHC: erythrocyte mean corpuscular hemoglobin concentration.

j PLT: platelet count.

k PCT: plateletocrit.

l MPV: mean platelet volume.

m LYMPH: lymphocyte count.

n LYM%: percentage of lymphocytes.

o PDW: platelet distribution width.

p HDL: high-density lipoprotein cholesterol.

q UA: urea acid.

r CRE: plasma creatine.

s GLU: glucose.

t TG: triglyceride.

u CHO: total cholesterol.

v CRPHS: high-sensitivity c-reactive protein.

w MDA: malondialdehyde.

x SOD: superoxide dismutase activity.

### Model Performance

**Figure 2.**
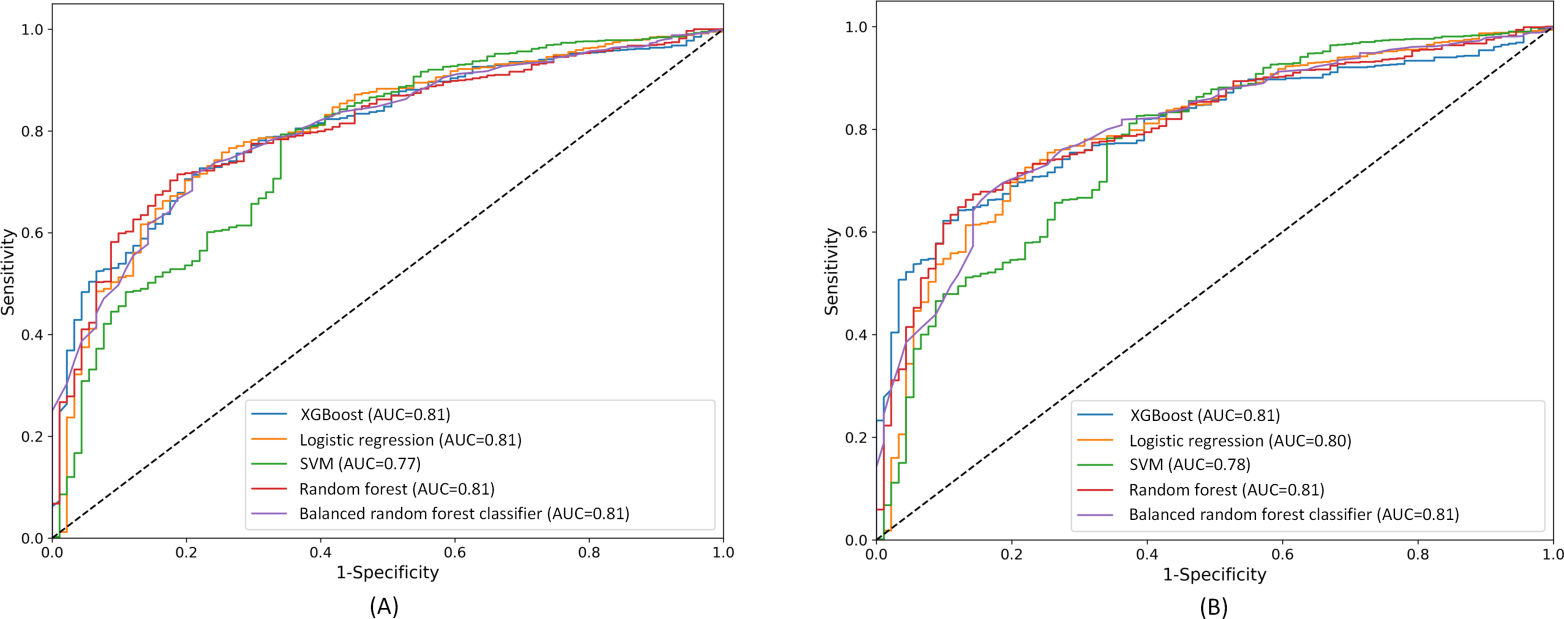
ROC curves with AUC values for machine learning models: (A) internal test set and (B) prospective validation set. AUC: area under the curve; ROC: receiver operating characteristic; SVM: support vector machines; XGBoost: Extreme Gradient Boosting.

The performance of the ML models in predicting follow-up cognitive impairment was evaluated using 5 algorithms—RF, XGBoost, logistic regression, SVM, and BRF—on both the internal test set and prospective validation set. Each model’s accuracy, sensitivity, and area under the receiver operating characteristic curve were assessed. The detailed results are presented in Table 2 and visually depicted in Figure 2. Additionally, we used the MMSE as an input for ML prediction. After testing several ML models, the overall performance remained suboptimal. For illustration, we selected 1 representative result, as shown in Table 2.

**Table 2.** The prediction results for ML^a^ models.

	Internal test			Prospective validation		
	Accuracy (95% CI)	Sensitivity (95% CI)	AUC^b^ (95% CI)	Accuracy (95% CI)	Sensitivity (95% CI)	AUC (95% CI)
RF^c^	0.828 (0.806-0.854)	0.688 (0.633-0.742)	0.81 (0.769-0.852)	0.813 (0.79-0.84)	0.684 (0.63-0.739)	0.806 (0.767-0.848)
XGBoost^d^	0.849 (0.825-0.87)	0.674 (0.621-0.728)	0.811 (0.769-0.851)	0.836 (0.812-0.857)	0.682 (0.629-0.733)	0.808 (0.768-0.848)
Logistic regression	0.778 (0.751-0.803)	0.715 (0.66-0.761)	0.803 (0.753-0.847)	0.771 (0.744-0.795)	0.745 (0.696-0.789)	0.804 (0.753-0.847)
SVM^e^	0.69 (0.661-0.719)	0.681 (0.629-0.731)	0.777 (0.725-0.827)	0.672 (0.643-0.701)	0.676 (0.623-0.725)	0.777 (0.726-0.826)
Balanced RF classifier	0.885 (0.866-0.903)	0.58 (0.54-0.626)	0.809 (0.765-0.849)	0.887 (0.867-0.905)	0.616 (0.572-0.664)	0.811 (0.767-0.849)
MMSE^f^	N/A^g^	N/A	0.571 (0.485-0.653)	N/A	N/A	0.558 (0.494-0.62)

a ML: machine learning.

b AUC: area under the curve.

c RF: random forest.

d XGBoost: Extreme Gradient Boosting.

e SVM: support vector machines.

f MMSE: Mini-Mental State Examination.

g N/A: not applicable.

In the internal test set, the BRF achieved the highest accuracy at 88.5% but had lower sensitivity at 58%. RF and XGBoost provided balanced results with accuracies of 82.8% and 84.9% and sensitivities of 68.8% and 67.4%, respectively. Logistic regression yielded a moderate accuracy of 77.8% but the highest sensitivity at 71.5%, while SVM had the lowest performance with 69% accuracy and 68.1% sensitivity. Similar patterns were observed in the prospective validation set, with BRF at 88.7% accuracy, logistic regression reaching 74.5% sensitivity, and SVM again showing the lowest overall performance. Additionally, the performance of all ML models was superior to that of the MMSE.

**Figure 3.**
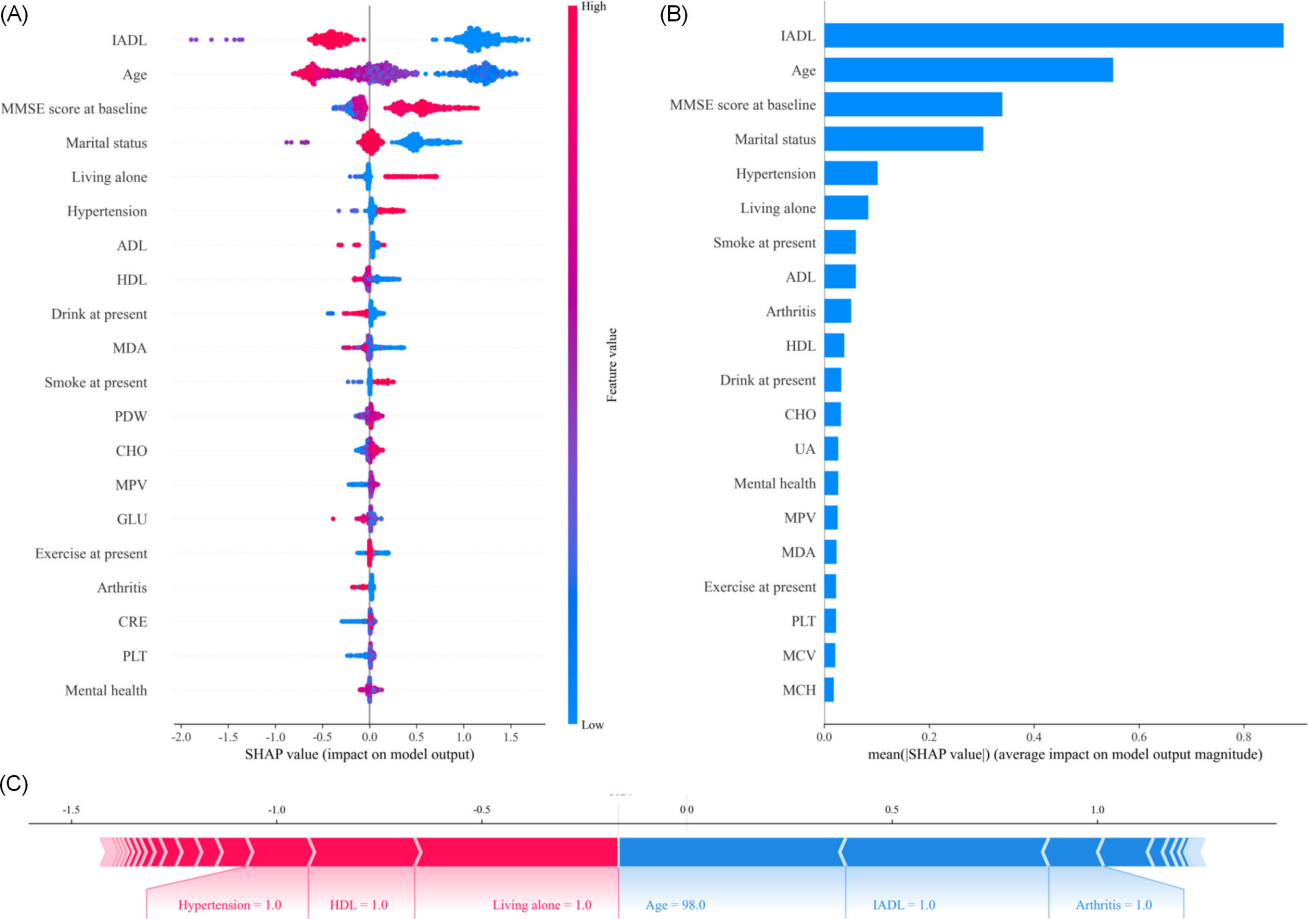
Explanation of the interpretability of the XGBoost (Extreme Gradient Boosting) model (the best-performing model) for predicting older adult mortality. (A) and (B) show the top 20 risk predictors for prediction of cognitive impairment subjects, and (C) shows the SHAP plots of a subject. ADL: activities of daily living; CHO: total cholesterol; CRE: plasma creatine; GLU: glucose; HDL: high-density lipoprotein cholesterol; IADL: instrumental activities of daily living; MCH: erythrocyte mean corpuscular hemoglobin; MCV: erythrocyte mean corpuscular volume; MDA: malondialdehyde; MMSE: Mini-Mental State Examination; MPV: mean platelet volume; PDW: platelet distribution width; PLT: platelet count; SHAP: Shapley Additive Explanations; UA: uric acid.

Figure 3 offers a detailed exposition of the XGBoost for cognitive impairment in older adult individuals, featuring the 20 most influential risk predictors. The model prioritizes IADL, age, and baseline MMSE scores as the top determinants, with marital status, living alone, and hypertension also providing significant predictive value. Other important factors include biological markers such as MDA and HDL, alongside lifestyle factors such as current exercise, smoking, and drinking habits.

Figure 3A illustrates the distribution of SHAP values for these predictors, indicating their impact on the XGBoost’s output. In this plot, each dot represents a single instance of a feature in the dataset, and the horizontal axis shows the SHAP value of that feature. The SHAP value indicates the contribution of each feature to the model’s prediction, with positive values suggesting that the feature increases the likelihood of cognitive impairment, and negative values indicating a decrease. The color of the dots, ranging from blue to red, represents the feature’s value, with blue corresponding to lower values and red indicating higher values of the predictor. This color scheme helps highlight how different values of each predictor influence the model’s outcome. A high SHAP value for a given feature corresponds to a high level of importance in the predictive model. Figure 3B features a bar plot that quantifies the average impact of each predictor, measured by the mean absolute SHAP value. The length of each bar represents the average contribution of a feature to the model’s output across all data points. Here, the most important features in the model—such as IADL, age, and MMSE score at baseline—are easily identified, as they have the longest bars, indicating that they have the highest average impact on the model’s predictions.

Figure 3C illustrates a specific case study, showing how the SHAP values for a particular individual (in this case, an adult aged 98 years) contribute to the XGBoost’s prediction of cognitive impairment. Each feature is shown with its value (eg, hypertension=1), and the arrows indicate how these values shift the model’s prediction. Features such as hypertension and living alone appear to have a red color, indicating they push the prediction toward a higher risk of cognitive impairment. Similarly, age (with a value of 98) and IADL further emphasize the risk in this individual’s profile. The interaction of these predictors is visualized through their SHAP values, which collectively guide the prediction model’s decision, offering an individualized risk profile for this person.

## Discussion

### Principal Findings

This study uses the capabilities of ML to integrate a diverse set of 39 predictors for forecasting the decline in cognitive function over a 3-year period, yielding significant findings that resonate with the existing body of literature. Our ML model underscores the importance of IADL, age, baseline MMSE scores, marital status, living arrangements, hypertension, arthritis, and general lifestyle habits as pivotal factors influencing cognitive function. These determinants are consistent with findings from prior research, thereby affirming the reliability and relevance of our analytical approach [12,22].

Consistent with prior studies, advanced age has been identified as a significant risk factor for cognitive impairment [35,36]. The risk of MCI in the older adults aged 65 years or older is as high as 10%‐20% [37]. The limitation of older adult individuals’ ability to perform activities, as assessed by ADL and IADL, restricts their range of activities, diminishes social interactions, and consequently reduces the cerebral stimulation necessary for maintaining cognitive functions [38,39]. Furthermore, the impact of living conditions on cognitive health is evidenced by the predictive value of marital status and living alone [22,40-42]. Older adult individuals residing with a spouse typically exhibit healthier brain functions due to increased communication and maintenance of a normal life.

Additionally, this study underscores the detrimental effects of unhealthy lifestyle behaviors such as smoking and drinking on cognitive function [43-45], as well as the risk of cognitive impairment caused by pre-existing health conditions [46-49]. For instance, an Indian study highlighted that older smokers were 24% more likely to experience cognitive impairment compared to nonsmokers [50]. Similarly, Sabia et al [45] reported that abstaining from alcohol or consuming more than 14 units per week in middle age escalates the risk of AD. Moreover, recent research suggests that up to 3% of dementia cases could be averted by enhancing physical activity levels [51,52]. Concerning health conditions, a community-based cohort study illustrated that hypertension is associated with an increased risk of both all-cause MCI and nonamnestic MCI, with hazard ratios of 1.4 and 1.7, respectively, after age and sex adjustments [49]. Additionally, Appenzeller et al [53] found that patients with rheumatoid arthritis exhibited a significantly higher incidence of cognitive impairment compared to healthy controls.

In this study, in addition to age, lifestyle behaviors, and disease history—which can be evaluated through questionnaires or scales—biomarkers were specifically included in the ML model to enhance the predictive accuracy of clinical risk assessments for cognitive decline. Prior research has indicated that count elevated levels of specific biomarkers, including MDA, HDL, platelets, mean platelet volume, platelet distribution width, mean corpuscular hemoglobin, CHO, lymphocyte percentage, and plasma creatinine, are associated with an increased risk of cognitive decline [54-65]. For clinicians, integrating a patient’s lifestyle behaviors with blood biochemical markers can aid in the assessment of cognitive function. For communities, these indicators can help identify residents who may be at high risk. For family members, this model enables the evaluation of older adult relatives who may be reluctant to acknowledge their cognitive decline, thereby facilitating timely medical intervention. The model developed in this study is versatile and offers valuable insights for the identification of cognitive impairment across various settings.

The ML model developed in this study has the potential to significantly improve clinical practice and primary care by providing a rapid, efficient, and accessible tool for identifying individuals at risk of cognitive decline. Traditional methods for cognitive function assessment, such as imaging techniques such as MRI, can be time-consuming and resource-intensive, especially in resource-limited settings. By leveraging routine data such as blood biomarkers, demographic information, and lifestyle factors, this model offers a cost-effective approach to identify individuals who may require further clinical evaluation or early intervention. In primary care settings, where health care professionals often manage large volumes of patients, the model can serve as a valuable screening tool to detect early cognitive decline and facilitate referrals for specialized care. Furthermore, by integrating this model into electronic health records, health care providers can make timely and informed decisions, improving patient outcomes through proactive management. In essence, the model has the potential to transform early detection and intervention strategies, shifting the focus toward preventative care and better allocation of health care resources.

Compared to previous studies using ML for cognitive impairment prediction, our study offers several distinct contributions. For example, while studies such as those by Hu et al [22] and Gao et al [66] have successfully developed ML models to predict cognitive impairment among Chinese community-dwelling older adult individuals, they often focused on a more limited set of predictors—typically emphasizing demographic factors and neuropsychological assessments. In contrast, our study integrates a comprehensive set of 39 predictors, including both routine blood biomarkers (eg, MDA and HDL) and detailed lifestyle and disease history data. This broader approach not only enhances predictive accuracy but also provides a rapid, cost-effective tool that can be easily applied in community and clinical settings. Moreover, while some previous work [67,68] has primarily relied on imaging data or traditional statistical methods, our use of advanced ensemble ML techniques (such as BRF and XGBoost) combined with SHAP-based interpretability offers a clearer understanding of individual risk factors. This interpretability is crucial for clinicians to tailor early intervention strategies. In summary, our study advances the field by delivering a more inclusive and interpretable model that effectively tracks cognitive decline over 3 years, thereby addressing gaps in existing research and offering tangible benefits for early detection and intervention.

Nevertheless, this study presents certain limitations that warrant consideration. Primarily, the dependence on MMSE scores as the sole measure of cognitive impairment may not fully represent the broad spectrum of cognitive health, as the MMSE mainly evaluates specific cognitive domains and does not address emotional or psychological aspects. Furthermore, there is potential bias due to the generally younger age and better overall function of this study’s participants compared to those who were lost to follow-up, which could skew the results. However, the representation of the dataset at a national level does provide a measure of balance, helping to partially offset these biases.

### Conclusions

In conclusion, this study validates the efficacy of a ML model integrating demographic data, lifestyle factors, and biomarkers to predict cognitive impairment in older adults. It underscores the significance of traditional risk factors such as age and daily functional abilities while highlighting the role of solitary living conditions and unhealthy habits in cognitive decline. By including a broad spectrum of biomarkers, the model enriches the predictive framework, offering clinicians, communities, and families a valuable tool for early identification and intervention in cognitive impairment, which could have far-reaching implications for public health and the well-being of the aging population.

### Limitations

While the CLHLS is a large-scale longitudinal study that primarily focuses on individuals aged 65 years and older, to assess the health status and longevity of the older adult population in China, it is important to acknowledge the limitations of the dataset concerning its representativeness. The CLHLS sample is designed to represent the health conditions of the older adult population in China, but it may not fully capture the global demographics of AD or other forms of dementia. Specifically, older adult populations in other countries or regions may differ by genetic background, lifestyle factors, and health risks, which could influence the development and progression of cognitive impairment. Future studies incorporating diverse, multinational cohorts would be beneficial in enhancing the generalizability and robustness of cognitive decline prediction models.

## Abbreviations

AD: Alzheimer disease
ADL: activities of daily living
BRF: balanced random forest
CHO: total cholesterol
CLHLS: Chinese Longitudinal Healthy Longevity Survey
HDL: high-density lipoprotein cholesterol
IADL: instrumental activities of daily living
MCI: mild cognitive impairment
MDA: malondialdehyde
ML: machine learning
MMSE: Mini-Mental State Examination
MRI: magnetic resonance imaging
RF: random forest
SHAP: Shapley Additive Explanations
SMOTE: Synthetic Minority Over-Sampling Technique
SVM: support vector machines
XGBoost: Extreme Gradient Boosting

## References

[1] Hane FT, Robinson M, Lee BY, Bai O, Leonenko Z, Albert MS (2017). Recent progress in Alzheimer’s disease research, part 3: diagnosis and treatment. J Alzheimers Dis.

[2] Scheltens P, De Strooper B, Kivipelto M (2021). Alzheimer’s disease. Lancet.

[3] (2023). 2023 Alzheimer’s disease facts and figures. Alzheimers Dement.

[4] Prince M, Wimo A, Ali GC, Wu YT, Prina M (2015). World Alzheimer report 2015: the global impact of dementia: an analysis of prevalence, incidence, cost and trends.

[5] Takizawa C, Thompson PL, van Walsem A, Faure C, Maier WC (2014). Epidemiological and economic burden of Alzheimer’s disease: a systematic literature review of data across Europe and the United States of America. JAD.

[6] Jia J, Wei C, Chen S (2018). The cost of Alzheimer’s disease in China and re-estimation of costs worldwide. Alzheimers Dement.

[7] Anderson ND (2019). State of the science on mild cognitive impairment (MCI). CNS Spectr.

[8] Tan CC, Yu JT, Tan L (2014). Biomarkers for preclinical Alzheimer’s disease. J Alzheimers Dis.

[9] Dong X, Nao J, Shi J, Zheng D (2019). Predictive value of routine peripheral blood biomarkers in Alzheimer’s disease. Front Aging Neurosci.

[10] Jia L, Du Y, Chu L (2020). Prevalence, risk factors, and management of dementia and mild cognitive impairment in adults aged 60 years or older in China: a cross-sectional study. Lancet Public Health.

[11] Jing F, Cheng M, Li J (2023). Social, lifestyle, and health status characteristics as a proxy for occupational burnout identification: a network approach analysis. Front Psychiatry.

[12] Tan WY, Hargreaves C, Chen C, Hilal S (2023). A machine learning approach for early diagnosis of cognitive impairment using population-based data. J Alzheimers Dis.

[13] Aguayo GA, Zhang L, Vaillant M (2023). Machine learning for predicting neurodegenerative diseases in the general older population: a cohort study. BMC Med Res Methodol.

[14] Tan M, Xiao Y, Jing F (2024). Evaluating machine learning-enabled and multimodal data-driven exercise prescriptions for mental health: a randomized controlled trial protocol. Front Psychiatry.

[15] Navarro CLA, Damen JAA, Takada T (2022). Completeness of reporting of clinical prediction models developed using supervised machine learning: a systematic review. BMC Med Res Methodol.

[16] Frizzell TO, Glashutter M, Liu CC (2022). Artificial intelligence in brain MRI analysis of Alzheimer’s disease over the past 12 years: a systematic review. Ageing Res Rev.

[17] Tombaugh TN, McIntyre NJ (1992). The Mini-Mental State Examination: a comprehensive review. J Am Geriatr Soc.

[18] Folstein MF, Folstein SE, McHugh PR (1975). “Mini-mental state”. J Psychiatr Res.

[19] (1998). The Chinese Longitudinal Healthy Longevity Survey (CLHLS)—Longitudinal Data (1998–2018). Peking University Open Research Data Platform.

[20] Zeng Y, Vaupel JW (2009). Chinese Longitudinal Healthy Longevity Survey (CLHLS), biomarkers datasets, 2009, 2012, 2014 (ICPSR 37226). National Archive of Computerized Data on Aging.

[21] Wu J, Li J (2018). The impact of social participation on older people’s death risk: an analysis from CLHLS. China Popul Dev Stud.

[22] Hu M, Shu X, Yu G, Wu X, Välimäki M, Feng H (2021). A risk prediction model based on machine learning for cognitive impairment among chinese community-dwelling elderly people with normal cognition: development and validation study. J Med Internet Res.

[23] Mlinac ME, Feng MC (2016). Assessment of activities of daily living, self-care, and independence. Arch Clin Neuropsychol.

[24] Farias ST, Mungas D, Reed BR, Harvey D, Cahn-Weiner D, Decarli C (2006). MCI is associated with deficits in everyday functioning. Alzheimer Dis Assoc Disord.

[25] Kiosses DN, Alexopoulos GS (2005). IADL functions, cognitive deficits, and severity of depression: a preliminary study. Am J Geriatr Psychiatry.

[26] Graf C (2008). The Lawton instrumental activities of daily living scale. AJN.

[27] Little RJA, Rubin DB. (2019). Statistical Analysis with Missing Data.

[28] Chawla NV, Bowyer KW, Hall LO, Kegelmeyer WP (2002). SMOTE: Synthetic Minority Over-sampling Technique. JAIR.

[29] Breiman L (2001). Random forests. Mach Learn.

[30] Chen T, Guestrin C XGBoost.

[31] Sturdivant RX, Hosmer DW, Lemeshow S (2013). Applied Logistic Regression.

[32] Cortes C, Vapnik V (1995). Support-vector networks. Mach Learn.

[33] Khalilia M, Chakraborty S, Popescu M (2011). Predicting disease risks from highly imbalanced data using random forest. BMC Med Inform Decis Mak.

[34] Mangalathu S, Hwang SH, Jeon JS (2020). Failure mode and effects analysis of RC members based on machine-learning-based Shapley Additive Explanations (SHAP) approach. Eng Struct.

[35] Cong L, Ren Y, Wang Y (2023). Mild cognitive impairment among rural-dwelling older adults in China: a community-based study. Alzheimers Dement.

[36] Grueso S, Viejo-Sobera R (2021). Machine learning methods for predicting progression from mild cognitive impairment to Alzheimer’s disease dementia: a systematic review. Alzheimers Res Ther.

[37] Langa KM, Levine DA (2014). The diagnosis and management of mild cognitive impairment: a clinical review. JAMA.

[38] Njegovan V, Hing MM, Mitchell SL, Molnar FJ (2001). The hierarchy of functional loss associated with cognitive decline in older persons. J Gerontol A Biol Sci Med Sci.

[39] Jekel K, Damian M, Wattmo C (2015). Mild cognitive impairment and deficits in instrumental activities of daily living: a systematic review. Alzheimers Res Ther.

[40] Sundström A, Westerlund O, Kotyrlo E (2016). Marital status and risk of dementia: a nationwide population-based prospective study from Sweden. BMJ Open.

[41] Sommerlad A, Ruegger J, Singh-Manoux A, Lewis G, Livingston G (2018). Marriage and risk of dementia: systematic review and meta-analysis of observational studies. J Neurol Neurosurg Psychiatry.

[42] Ahn IS, Kim JH, Kim S (2009). Impairment of instrumental activities of daily living in patients with mild cognitive impairment. Psychiatry Investig.

[43] Tyas SL, White LR, Petrovitch H (2003). Mid-life smoking and late-life dementia: the Honolulu-Asia aging study. Neurobiol Aging.

[44] Xu W, Wang H, Wan Y (2017). Alcohol consumption and dementia risk: a dose-response meta-analysis of prospective studies. Eur J Epidemiol.

[45] Sabia S, Fayosse A, Dumurgier J (2018). Alcohol consumption and risk of dementia: 23 year follow-up of Whitehall II cohort study. BMJ.

[46] Vitturi BK, Nascimento BAC, Alves BR, de Campos FSC, Torigoe DY (2019). Cognitive impairment in patients with rheumatoid arthritis. J Clin Neurosci.

[47] Meade T, Manolios N, Cumming SR, Conaghan PG, Katz P (2018). Cognitive impairment in rheumatoid arthritis: a systematic review. Arthritis Care Res (Hoboken).

[48] Aronow WS (2017). Hypertension and cognitive impairment. Ann Transl Med.

[49] Reitz C, Tang MX, Manly J, Mayeux R, Luchsinger JA (2007). Hypertension and the risk of mild cognitive impairment. Arch Neurol.

[50] Muhammad T, Govindu M, Srivastava S (2021). Relationship between chewing tobacco, smoking, consuming alcohol and cognitive impairment among older adults in India: a cross-sectional study. BMC Geriatr.

[51] Liang JH, Lu L, Li JY (2020). Contributions of modifiable risk factors to dementia incidence: a Bayesian network analysis. J Am Med Dir Assoc.

[52] Dominguez LJ, Veronese N, Vernuccio L (2021). Nutrition, physical activity, and other lifestyle factors in the prevention of cognitive decline and dementia. Nutrients.

[53] Appenzeller S, Bertolo MB, Costallat LTL (2004). Cognitive impairment in rheumatoid arthritis. Methods Find Exp Clin Pharmacol.

[54] Keller JN, Schmitt FA, Scheff SW (2005). Evidence of increased oxidative damage in subjects with mild cognitive impairment. Neurology (ECronicon).

[55] Liu Z, Liu Y, Tu X (2017). High serum levels of malondialdehyde and 8-OHdG are both associated with early cognitive impairment in patients with acute ischaemic stroke. Sci Rep.

[56] van Exel E, de Craen AJM, Gussekloo J (2002). Association between high-density lipoprotein and cognitive impairment in the oldest old. Ann Neurol.

[57] Atzmon G, Gabriely I, Greiner W, Davidson D, Schechter C, Barzilai N (2002). Plasma HDL levels highly correlate with cognitive function in exceptional longevity. J Gerontol A Biol Sci Med Sci.

[58] Crichton GE, Elias MF, Davey A, Sullivan KJ, Robbins MA (2014). Higher HDL cholesterol is associated with better cognitive function: the Maine-Syracuse study. J Int Neuropsychol Soc.

[59] Zhang Y, Liu J, Wei Z (2023). Elevated serum platelet count inhibits the effects of brain functional changes on cognitive function in patients with mild cognitive impairment: A resting-state functional magnetic resonance imaging study. Front Aging Neurosci.

[60] Sun D, Wang Q, Kang J (2021). Correlation between serum platelet count and cognitive function in patients with atrial fibrillation: a cross-sectional study. Cardiol Res Pract.

[61] Wang RT, Jin D, Li Y, Liang QC (2013). Decreased mean platelet volume and platelet distribution width are associated with mild cognitive impairment and Alzheimer’s disease. J Psychiatr Res.

[62] Winchester LM, Powell J, Lovestone S, Nevado-Holgado AJ (2018). Red blood cell indices and anaemia as causative factors for cognitive function deficits and for Alzheimer’s disease. Genome Med.

[63] Guo Y, Li P, Ma X (2020). Association of circulating cholesterol level with cognitive function and mild cognitive impairment in the elderly: a community-based population study. Curr Alzheimer Res.

[64] Magaki S, Yellon SM, Mueller C, Kirsch WM (2008). Immunophenotypes in the circulation of patients with mild cognitive impairment. J Psychiatr Res.

[65] Yoshida M, Higashi K, Kuni K (2015). Distinguishing mild cognitive impairment from Alzheimer’s disease with acrolein metabolites and creatinine in urine. Clinica Chimica Acta.

[66] Gao H, Schneider S, Hernandez R (2024). Early identification of cognitive impairment in community environments through modeling subtle inconsistencies in questionnaire responses: machine learning model development and validation. JMIR Form Res.

[67] Graham SA, Lee EE, Jeste DV (2020). Artificial intelligence approaches to predicting and detecting cognitive decline in older adults: a conceptual review. Psychiatry Res.

[68] Zhou Y, Song Z, Han X, Li H, Tang X (2021). Prediction of Alzheimer’s disease progression based on magnetic resonance imaging. ACS Chem Neurosci.

